# Short-Term Recovery Interventions Using Cryosauna, Cold-Water Immersion, and Foam Rolling in Mixed Martial Arts Athletes: A Polish Pilot Study

**DOI:** 10.3390/sports14060244

**Published:** 2026-06-12

**Authors:** Behnam Boobani, Juris Grants, Hubert Makaruk, Dariusz Gierczuk, Tomasz Sacewicz, Marcin Starzak, Žermēna Vazne, Tatjana Glaskova-Kuzmina, Artur Litwiniuk

**Affiliations:** 1 RSU Latvian Academy of Sport Education, Riga Stradins University, LV-1006 Riga, Latvia; juris.grants@rsu.lv; 2 Faculty of Physical Education and Health, Jozef Pilsudski University of Physical Education in Warsaw, 21-500 Biala Podlaska, Poland; hubert.makaruk@awf.edu.pl (H.M.); dariusz.gierczuk@awf.edu.pl (D.G.); tomasz.sacewicz@awf.edu.pl (T.S.); marcin.starzak@awf.edu.pl (M.S.); a.litwiniuk@wp.pl (A.L.); 3 Department of Health Psychology and Pedagogy, Riga Stradins University, LV-1007 Riga, Latvia; zermena.vazne@rsu.lv; 4 Faculty of Science and Technology, University of Latvia, LV-1004 Riga, Latvia; tatjana.glaskova-kuzmina@lu.lv

**Keywords:** performance enhancement, athletes, combat sports, recovery modalities, cognitive and physical testing

## Abstract

Background: Mixed martial arts (MMA) involve repeated high-intensity, explosive actions that cause substantial fatigue, underscoring the importance of effective recovery strategies. Purpose: This pilot study investigated short-term performance responses to different post-exercise recovery interventions in Polish MMA athletes. Methods: Sixteen athletes (14 males and 2 females) were randomly assigned to cryosauna (CRYO), cold-water immersion (CWI), foam rolling (FR), or passive recovery (CON), with 4 participants per group. The intervention lasted two weeks, with the assigned recovery intervention applied after each training session. Performance was evaluated before and after the intervention using the countermovement jump (CMJ), isokinetic knee peak torque (flexion and extension), and reactive stress tolerance of the determination test (DT). Data were analyzed using mixed-design ANOVA. Results: CMJ performance improved over time across groups. FR significantly increased knee extension (from 228.67 ± 26.49 N.m to 250.50 ± 22.41 N.m), whereas DT scores significantly increased in the CRYO group (from 247.50 ± 12.50 AU to 291.50 ± 15.61 AU) and significantly decreased in the CON group (from 290.25 ± 24.45 AU to 255.50 ± 24.18 AU). Significant Time × Group interactions were observed for DT (*p* < 0.001) and knee extension torque (*p* = 0.008). Conclusions: FR appeared beneficial for knee extension performance, whereas CRYO was associated with improved DT performance. Findings are exploratory and need confirmation in larger, controlled studies.

## 1. Introduction

The increasing physical demands placed on athletes at all levels have heightened the importance of sports science research, not only to optimize training processes but also to improve recovery between training sessions and competitive events. Adequate recovery is a key component of athletic preparation, as accumulated fatigue can negatively affect subsequent performance and reduce overall physical readiness [1]. Martial arts are characterized by high-intensity, intermittent activity patterns that frequently result in multiple forms of fatigue. These fatigue responses are a major factor contributing to performance deterioration and increased recovery requirements [2]. Accordingly, implementing effective recovery strategies is critical for minimizing fatigue-induced stress, reducing injury risk, and maintaining stable performance throughout training and competitive periods [3]. A variety of post-exercise recovery methods have gained attention in recent years, including external cooling techniques and foam rolling interventions [4], cold water immersion and blood flow restriction [5,6], the use of contrast therapy [7,8], and many other strategies.

Among these, cryosauna (CRYO) has emerged as a commercially available and increasingly used modality. This approach involves brief exposure (approximately 1–3 min) to extremely low temperatures generated by liquid nitrogen vapor, typically ranging from −110 °C to −195 °C, while individuals wear minimal protective clothing [9]. Cryotherapy may help reduce inflammation and pain and attenuate muscle soreness and exercise-induced muscle damage through cold-related analgesic effects [10]. In parallel, cold-water immersion (CWI) remains one of the most widely used recovery strategies in athletic settings, typically using water temperatures between 5 °C and 13 °C for approximately 10–24 min [11]. CWI may enhance recovery by improving lactate removal and reducing post-exercise thermal and cardiovascular strain via cooling-induced vasoconstriction [12,13]. In addition to conventional massage techniques, foam rolling has become widely used in sports and fitness settings and is now one of the most frequently used recovery methods among athletes [14,15]. Massage can support recovery by reducing psychological stress and promoting relaxation after exercise, though its effectiveness depends on the technique used and individual responses. Despite the growing popularity of cold-based interventions and foam rolling across a wide range of sports, including martial arts, uncertainty remains regarding the most effective cooling protocols and their influence on post-exercise performance recovery [4,5,6,7,8,9,11,13,14,15,16,17,18,19]. While some evidence suggests that partial-body cryotherapy, cold-water immersion, and foam rolling may enhance jump performance and peak force outcomes compared with passive recovery, findings across studies remain inconsistent [4,19].

Conversely, several investigations have reported reductions in countermovement jump performance following cryotherapy in martial arts athletes, suggesting that acute cold exposure may transiently suppress force-generating capacity due to lowered muscle temperature [20,21,22]. These divergent findings appear strongly context-dependent and are shaped by factors such as sport-specific demands, training intensity, selected performance outcomes, and the timing of assessment [23]. Although the application of appropriate recovery strategies is crucial for maintaining high-level performance and reducing the adverse effects of fatigue, empirical evidence on recovery practices among martial arts athletes remains scarce [24]. Accordingly, this study was conducted as an exploratory pilot investigation to evaluate short-term performance responses to several post-exercise recovery approaches, including cryosauna (CRYO), cold-water immersion (CWI), foam rolling (FR), and passive recovery (Con), in Polish martial arts athletes. It was hypothesized that these recovery strategies would produce differential effects on countermovement jump performance (CMJ), isokinetic knee peak torque during flexion and extension, and reactive stress tolerance measured by the determination test (DT).

## 2. Materials and Methods

### 2.1. Participants

Sixteen mixed martial arts (MMA) athletes (2 females, 14 males) from Biala Podlaska, Poland, volunteered to participate in the study (Table 1). In line with the exploratory pilot design, the sample size was primarily determined by feasibility and the availability of eligible athletes during the study period. Participants were recruited through announcements distributed at local martial arts clubs and training facilities. Consistent with the exploratory pilot design, the sample was small and heterogeneous; therefore, no a priori or post hoc power analyses were conducted. Inclusion criteria required participation in national-level competitions, a minimum of five years of training experience, and regular training at least four times per week. Athletes were excluded if they reported current medication use, cold intolerance, cardiovascular or neurological disorders, or musculoskeletal injuries. Participants had at least two years of competitive experience and generally represented light- to middle-weight categories (~65–89 kg). Previous experience with recovery strategies was not considered an inclusion or exclusion criterion. Participants were instructed to maintain their usual diet, sleep, and training routines and to avoid extreme exercises, alcohol, and caffeine for 24 h before testing. No athlete reported rapid weight-loss practices or recent competition participation during the study period. Descriptive characteristics of participants are shown in Table 1. Anthropometric measurements were obtained using standardized equipment (Seca GmbH 213 and 813, Hamburg, Germany). The study was performed in accordance with the Declaration of Helsinki and approved by the Ethical Committee of the Józef Piłsudski University of Physical Education in Warsaw (No. SKE.0030.67.2025). Written informed consent was obtained from all participants.

### 2.2. Study Design

**Figure 1:**
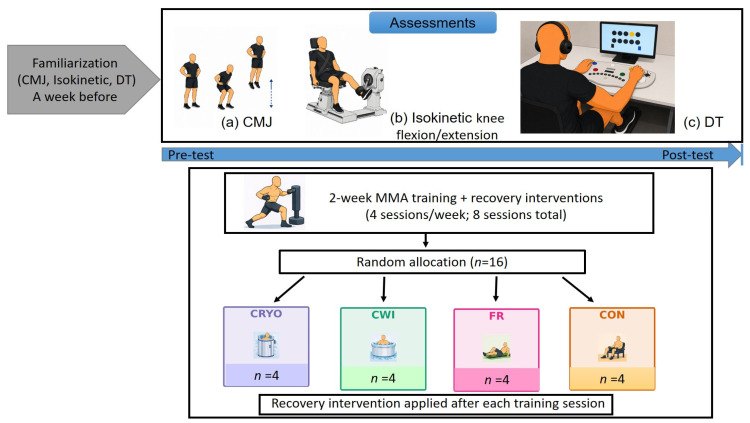
Study design flowchart. CMJ: countermovement jump; DT: determination test; CRYO: cryosauna; CWI: cold-water immersion; FR: foam rolling; CON: control group.

The study employed a pre–post experimental design with baseline and post-intervention assessments separated by two weeks (Figure 1). Participants were familiarized with all testing and recovery procedures one week before baseline testing to ensure understanding and minimize potential learning effects during performance testing. During the familiarization session, participants completed three practice countermovement jumps (CMJs), three submaximal repetitions of the isokinetic knee flexion–extension test, and a brief one-minute practice trial of the Determination Test (DT). On the experimental day, athletes were randomly assigned to cryosauna (CRYO), cold-water immersion (CWI), foam rolling (FR), or passive recovery (CON). Sixteen numbered allocations (four per intervention condition) were prepared and randomly shuffled in advance by a researcher not involved in performance testing. Each participant then selected one concealed number to determine group assignment, resulting in equal group sizes (*n* = 4 per group). After baseline testing, athletes continued training for two weeks (eight sessions, four per week), with the designated recovery intervention applied after each session. Performance assessments at both time points included countermovement jump (CMJ), isokinetic knee peak torque (flexion and extension), and reactive stress tolerance measured by the determination test (DT). All testing and recovery sessions were conducted between 15:00 and 17:00 to standardize testing conditions and ensure consistency across all experimental sessions, which corresponded to the schedule of regular training sessions for the athletes at the Józef Piłsudski University of Physical Education branch in Biala Podlaska, Poland, by the same investigator, who was not blinded to group allocation.

### 2.3. Recovery Interventions

During the two-week intervention (eight training sessions), athletes assigned to the cryosauna (CRYO) group completed 3 min of exposure to vaporized liquid nitrogen at −110 °C using a cryo-cabin (Juka, Niepołomice, Poland) following each session [19]. Participants removed metal objects and wore protective gloves and footwear while standing upright with the head positioned outside the chamber. The cold-water immersion (CWI) group underwent seated immersion (waist-level) at 10 °C for 20 min, with the water temperature continuously monitored and participants instructed to move their legs intermittently to ensure uniform cooling [25]. The foam rolling (FR) group performed self-myofascial release using a compact foam roller (Sveltus, Le Chambon-Feugerolles, France), targeting the quadriceps, hamstrings, adductors, and gastrocnemius. Three 30 s sets per muscle group were performed with 30 s rest intervals using controlled rolling movements [26]. To standardize movement cadence, an online metronome was set to 60 beats per minute. Participants performed a single rolling movement (either upward or downward along the muscle) for each metronome signal. The control group performed passive seated recovery for 20 min, consistent with previous control conditions [25].

### 2.4. Training Program

The training program (Table 2) remained unchanged throughout the study and was performed four times per week over two weeks (eight sessions, 90 min each). Sessions included a standardized warm-up, technical and sparring drills, and plyometric exercises (60 cm box jumps) [19]. During the plyometric exercise, participants stepped off the box, immediately executed a maximal vertical jump upon landing, and then returned to the ground. They were instructed to keep their hands on their hips and to land with about 90° of knee flexion. Box jumps are commonly incorporated into MMA training to develop explosive lower-limb performance [6]. Training intensity was maintained at approximately 85% of predicted maximal heart rate, monitored by pulse oximetry and estimated using Tanaka’s age-based equation (Max HR = 208 − (0.7 × age)) [27].

### 2.5. Performance Testing

#### 2.5.1. CMJ

Participants performed vertical jumps with hands placed on the hips and a standardized countermovement depth of approximately 90° knee flexion under verbal instruction [3]. Three trials were performed with 15 s rest intervals, consistent with previous CMJ monitoring protocols using short inter-trial recovery periods [28], and the highest jump height was retained for analysis. CMJ performance was assessed using the OptoJump system (Microgate^®^, Bolzano, Italy), which demonstrates excellent test–retest reliability (ICC = 0.98) [29].

#### 2.5.2. Isokinetic Peak Torque of the Knee (Flexion and Extension)

Knee flexion and extension peak torque were tested on a Biodex System 4 Pro dynamometer (Biodex Medical Systems, Shirley, NY, USA) following manufacturer calibration. Athletes completed a 10 min warm-up at 50 W on a cycle ergometer and stretching of the quadriceps and hamstrings for 5 min. Athletes were positioned and firmly strapped (trunk, hips, and thigh). The rotational axis of the dynamometer was aligned with the lateral femoral epicondyle, and the leg was securely attached to the lever arm just above the ankle using a padded cuff. The dominant leg was defined as the self-reported kicking leg, which was tested in all participants [30]. Isokinetic concentric/concentric (CON/CON) knee flexion and extension were performed at an angular velocity of 60°·s^−1^ through a range of motion of approximately 10–100°. Participants completed five maximal contractions under standardized verbal encouragement, with a 60 s rest interval between testing sets [31]. This protocol provides good-to-excellent reliability for knee strength outcomes (ICC > 0.87) [32].

#### 2.5.3. Determination Test

The determination test is a computerized assessment commonly used to evaluate cognitive performance and reaction-stress tolerance in sport settings [27]. During the test, participants were seated in front of the monitor and response console, exposed to both visual and auditory stimuli, and instructed to respond as rapidly and accurately as possible using the response console. Visual cues consisted of five colored signals (white, yellow, red, blue, and green) displayed at different positions on the monitor, along with left- and right-side foot indicators (Figure 1). Participants responded to these stimuli by pressing the matching colored hand button or the corresponding foot pedal. Auditory cues included high- and low-frequency tones delivered via the system interface, requiring participants to press either the upper gray rectangular button or the lower black rectangular button at the center of the response console. The total number of correct responses was recorded and included in the statistical analysis. These demands are relevant to MMA competition, where athletes must rapidly perceive external cues, make decisions under time pressure, and execute appropriate actions while managing high levels of cognitive and emotional stress [27]. Accordingly, DT outcomes were selected as indicators of performance readiness, reflecting decision-making under pressure and stress tolerance relevant to MMA performance. Test–retest reliability of the DT has been reported as good (r = 0.90 over 7–14 days) [31]. A minimum of 3 min of passive rest was provided between different performance tests to minimize fatigue-related effects [33]. All assessments were conducted between 15:00 and 17:00 in the laboratory of the Józef Piłsudski University of Physical Education branch in Biala Podlaska, Poland.

### 2.6. Statistical Analysis

Data are reported as means ± standard deviations. Percentage change (% change) scores were calculated as [(post − pre)/pre × 100]. Normality and homogeneity of variance were assessed using the Shapiro–Wilk test, visual inspection of Q–Q plots (Supplementary Figures S1–S4), and Levene’s test, respectively. Performance variables were analyzed using a 2 (Time: pre, post) × 4 (Group: CRYO, CWI, FR, CON) mixed-design ANOVA, with Bonferroni-adjusted post hoc tests applied when appropriate. When the assumption of homogeneity was violated, between-group differences in change scores (post − pre) were additionally examined using Welch-corrected one-way ANOVA. Effect sizes are presented as partial eta squared (η^2^p) and interpreted as small (≥0.01), medium (≥0.06), or large (≥0.14) [34]. Schematic and graphical figures were generated using Python (version 3.13.9) and Matplotlib (version 3.10) within a Jupyter Notebook environment (version 7.4). All analyses were conducted in JASP (version 0.18.3), with statistical significance set at *p* < 0.05.

## 3. Results

Descriptive statistics, % change, and 95% confidence intervals for all groups are provided in the Supplementary Materials (Table S1).

### 3.1. Countermovement Jump

**Figure 2:**
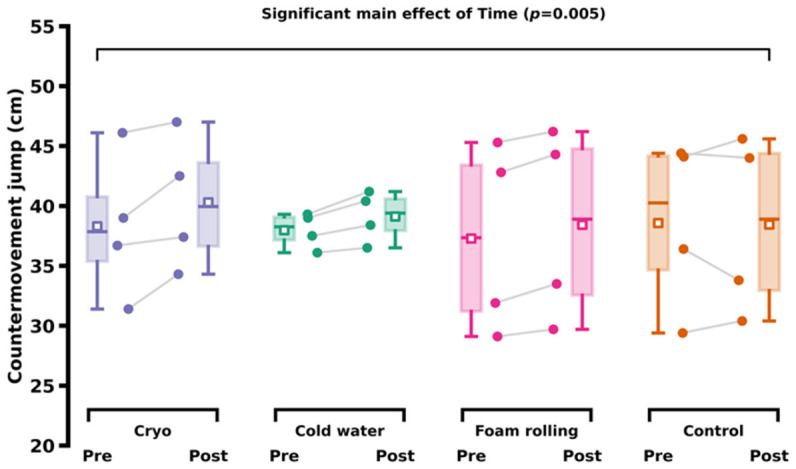
Pre- and post-test countermovement jump (CMJ) performance across the cryosauna (CRYO), cold-water immersion (CWI), foam rolling (FR), and control (CON) groups.

Mean CMJ performance increased from pre- to post-test in the CRYO (+5.22%), CWI (+3.02%), and FR (+3.08%) groups, whereas the CON group showed minimal change (−0.33%). Consistent with descriptive findings, a significant main effect of Time (Figure 2) indicated an overall improvement from pre- to post-test (F (1,12) = 11.61, *p* = 0.005, η^2^p = 0.49). Neither the Time × Group interaction (F (3,12) = 2.04, *p* = 0.16) nor the main effect of Group (F (3,12) = 0.03, *p* = 0.99) reached significance. Welch’s test showed no between-group differences in CMJ change scores (F (3,6.15) = 0.91, *p* = 0.48).

### 3.2. Isokinetic Peak Torque of the Knee (Flexion and Extension)

**Figure 3:**
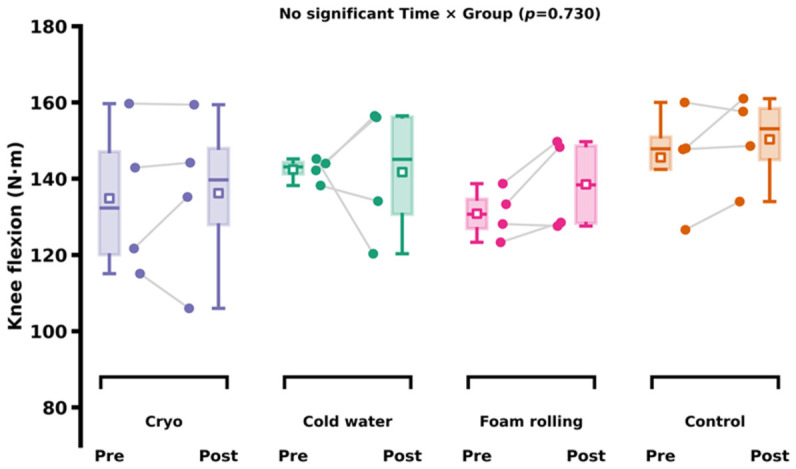
Pre- and post-test knee flexion peak torque across the cryosauna (CRYO), cold-water immersion (CWI), foam rolling (FR), and control (CON) groups.

Mean knee flexion peak torque did not significantly change from pre- to post-test, with slight increases in the CRYO (+0.96%), FR (+5.80%), and CON (+3.22%) groups, and a slight decrease in the CWI group (−0.42%). No significant Time × Group interaction (Figure 3) was observed (F (3,12) = 0.42, *p* = 0.73, η^2^p = 0.09). Neither the main effect of Time (F (1,12) = 1.35, *p* = 0.26) nor Group (F (3,12) = 0.81, *p* = 0.51) was significant, and Welch’s test confirmed no between-group differences in change scores (F (3,6.46) = 0.45, *p* = 0.72).

**Figure 4:**
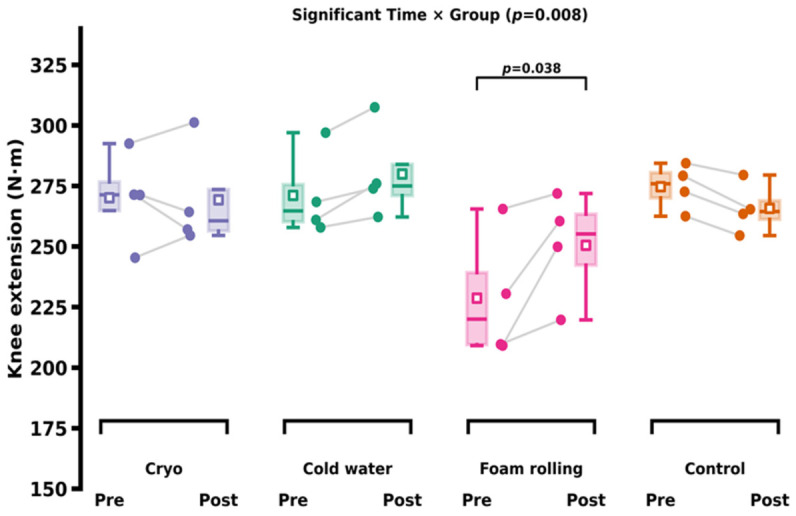
Pre- and post-test knee extension peak torque across the cryosauna (CRYO), cold-water immersion (CWI), foam rolling (FR), and control (CON) groups.

Mean knee extension peak torque increased significantly in the FR group (+9.53%), whereas no significant changes were observed in the CRYO (−0.33%), CWI (+3.25%), or CON (−3.28%) groups from pre- to post-test. A significant Time × Group interaction (F (3,12) = 6.34, *p* = 0.008, η^2^p = 0.61) was observed, indicating recovery-specific response patterns (Figure 4). The main effects of Time (F (1,12) = 3.92, *p* = 0.07) and Group (F (3,12) = 3.14, *p* = 0.06) did not reach statistical significance. Post hoc analysis revealed a significant pre- to post-test increase in knee extension torque in the FR group (*p* = 0.038), while no significant changes were observed in the other groups.

### 3.3. Determination Test

**Figure 5:**
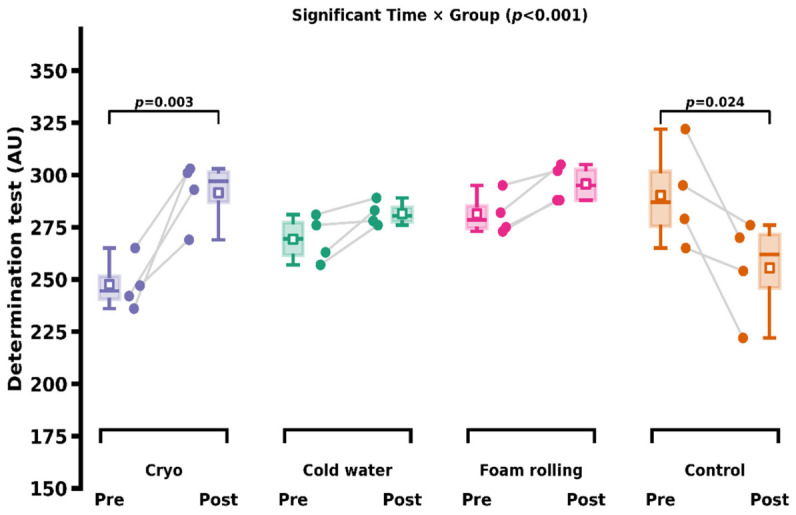
Pre- and post-test determination test across the cryosauna (CRYO), cold-water immersion (CWI), foam rolling (FR), and control (CON) groups, AU: arbitrary units.

Mean DT performance increased significantly in the CRYO group (+17.77%) and decreased significantly in the CON group (−11.98%), whereas no significant changes were observed in the CWI (+4.53%) or FR (+5.15%) groups from pre- to post-test. A significant Time × Group interaction (F (3,12) = 17.10, *p* < 0.001, η^2^p = 0.81) was observed indicating recovery-specific response patterns (Figure 5). A significant main effect of Time was also identified (F (1,12) = 5.22, *p* = 0.04, η^2^p = 0.30), whereas the main effect of Group was not significant (F (3,12) = 1.55, *p* = 0.25). Post hoc analyses revealed a significant pre- to post-test increase in DT scores in the CRYO group (*p* = 0.003) and a significant decrease in the CON group (*p* = 0.024), with no significant changes observed in the CWI or FR groups.

## 4. Discussion

This pilot study investigated short-term performance responses to cryosauna, cold-water immersion, foam rolling, and passive recovery in Polish mixed martial arts athletes. Results showed overall time-related improvements in CMJ and DT outcomes. Significant Time × Group interactions indicated that FR significantly increased knee extension torque, whereas CRYO improved DT. In contrast, DT performance decreased in the control group, whereas knee flexion torque remained unchanged across interventions. However, given the exploratory design and small sample size, these findings should be interpreted cautiously and require confirmation in larger studies.

Countermovement jump height reflects recovery of lower-limb explosive power and neuromuscular function. In the present study, CMJ performance improved over time; however, no differences were detected between recovery groups. The overall time-related improvement may partly reflect short-term neuromuscular adaptations associated with repeated plyometric training throughout the intervention period. Box jumps are commonly incorporated into MMA training to develop explosive lower-limb performance [6]. This aligns with previous findings showing that cold-water immersion and cryotherapy elicit pronounced physiological and perceptual responses but do not consistently enhance functional outcomes, such as jump performance, compared with passive recovery [16]. Although some studies report CMJ improvements following shorter cryotherapy or cold-water immersion protocols and cooler water temperatures (5–10 °C), results remain inconsistent and appear highly dependent on protocol characteristics and athlete population [2,4,11,16,35]. Prolonged or colder cooling exposures may increase muscle stiffness or peripheral vasoconstriction, potentially limiting neuro-muscular benefits [36]. Accordingly, neither cryosauna nor cold-water immersion provided additional CMJ benefits beyond the general time-related improvements observed across all groups. Similarly, foam rolling did not produce recovery-specific CMJ gains under the present conditions. While individual factors such as sex may influence foam-rolling responses [15,37], the small, unbalanced sample precluded evaluation of these effects in this pilot study.

Knee flexion torque remained unchanged throughout the intervention. In contrast, foam rolling significantly increased knee extension torque, whereas the other recovery interventions did not produce significant changes. In contrast to previous studies reporting limited effects of massage-based recovery methods on peak torque [38], foam rolling in the present study significantly improved knee extension torque after repeated MMA training sessions. Nevertheless, findings on foam rolling and muscle torque recovery remain inconsistent across studies [38,39]. These discrepancies may partly reflect differences in rolling protocols, applied pressure, training status, and outcome measures [39]. Cold-water immersion showed only a small, non-significant increase; cryosauna showed minimal change; and the control group’s values slightly decreased. These findings are consistent with previous reports showing limited effects of cryotherapy on knee muscle force recovery following eccentric exercise [17,40]. Cooling interventions may temporarily reduce muscular force output by affecting muscle contractile properties, reducing nerve conduction efficiency, and altering physiological mechanisms involved in muscle adaptation and recovery [22]. Variability in responses may be influenced by factors such as the cooling modality (whole- vs. partial-body), exposure duration, and the nature of the exercise stimulus. Cold-based recovery methods, including CWI, show inconsistent effects on force and torque production [41,42]. Acute cooling may reduce isokinetic torque [39], while other studies report differential effects depending on contraction mode, with preserved or improved isometric strength but reduced concentric torque [42]. Such variability likely reflects differences in cooling application and movement characteristics, which may explain the heterogeneous knee strength responses observed in the present study.

DT performance showed significant time-dependent changes, with DT scores improving significantly after cryosauna, whereas the control group showed a significant decline. Unlike earlier findings that predominantly reported familiarization-driven improvements [4], the present results indicate distinct DT responses across recovery conditions. Similar mixed cognitive responses to repeated cold exposure, influenced by arousal and attentional mechanisms, have been reported previously [43]. The positive DT response observed after cryosauna may suggest a beneficial effect of cold exposure on cognitive recovery and reactive stress tolerance following repeated MMA training sessions.

The present findings are limited by the relatively small sample size and pilot design, which restrict statistical power and generalizability. In addition, the absence of objective physiological recovery markers, detailed body composition assessment (e.g., fat and muscle mass), and limited control over external factors such as sleep, nutrition, hydration status, and training load may have contributed to individual variability [44]. The selected performance tests were also not fully sport-specific to MMA competition, and athletes’ primary striking- or grappling-oriented backgrounds were not formally categorized. Foam rolling cadence was standardized using a metronome (60 beats per minute), which may differ from the self-selected tempo commonly used in practice. Another limitation of the present pilot study is the small number of female participants (*n* = 2), which reduces the sample’s representativeness and limits the feasibility of meaningful sex-specific analyses. Finally, although a parallel-group design was used in the present pilot study, a crossover design may offer greater statistical sensitivity and reduce inter-individual variability in future studies with small sample sizes. Therefore, the present findings should be interpreted as exploratory, and future studies should incorporate larger samples and placebo-controlled designs.

## 5. Conclusions

This pilot study investigated short-term post-exercise recovery strategies in Polish MMA athletes. Countermovement jump performance and reactive stress tolerance improved over time, while knee flexion torque remained unchanged. Foam rolling significantly improved knee extension torque, whereas cryosauna was associated with improved DT performance. In contrast, DT performance declined in the passive recovery group. These findings suggest modality-specific responses to post-exercise recovery interventions; however, given the exploratory pilot design and small sample size, the results should be interpreted cautiously. Larger, well-controlled studies incorporating physiological and neurocognitive markers are needed to better define recovery-specific effects in mixed martial arts athletes.

## Figures and Tables

**Table 1:** Descriptive characteristics of Participants.

	Group (*n* = 16)				
	Cryo *n* = 4	CWI *n* = 4	FR *n* = 4	Control *n* = 4	*p*-Value
Age (years)	21.25 ± 1.25	22.50 ± 2.64	22.00 ± 2.94	22.25 ± 2.21	0.88
Height (cm)	178.25 ± 8.80	182.25 ± 2.63	174.25 ± 7.58	176.25 ± 2.06	0.32
Weight (kg)	77.00 ± 11.42	80.75 ± 7.21	71.12 ± 4.66	73.95 ± 4.44	0.33
Body mass index (kg/m^2^)	24.15 ± 2.36	24.31 ± 2.03	23.47 ± 1.11	23.84 ± 1.11	0.90
Training experience (years)	6.5 ± 1.29	7 ± 1.63	6.25 ± 1.50	6.75 ± 0.95	0.87

Note: values are means ± standard deviation. cm: centimeter; kg: kilogram.

**Table 2:** MMA training session used during the intervention period.

Training Phase	Exercise Content	Volume/Duration	Rest Interval
Warm-up	Dynamic mobility exercises, joint activation, light shadow boxing	~15 min	–
Technical striking drills	Punching and kicking combinations, pad work, and footwork drills	~10 min	~60–120 s between drill blocks
Grappling drills	Clinch techniques, takedown practice, and ground control drills	~20 min	~60–120 s between drill blocks
Sparring	Controlled MMA sparring rounds, integrating striking and grappling exchanges	~15 min	~60 s between rounds
Plyometric exercises	Box jumps (60 cm)	3–4 sets × 2–4 repetitions	~90 s between sets
Cool-down/transition	Light movement before recovery intervention	~5 min	–

Note. Training intensity was maintained at approximately 85% of predicted maximal heart rate.

## Data Availability

Data are available upon request to the correspondence author.

## Supplementary Materials

The following supporting information can be downloaded at https://www.mdpi.com/article/10.3390/sports14060244/s1, Supplementary Figure S1. Q–Q plots for assessing the normality of countermovement jump (CMJ) data, Figure S2. Q–Q plots are used for the graphical assessment of normality for the isokinetic peak torque of the knee (flexion) data, Figure S3. Q–Q plots are used for the graphical assessment of normality for the isokinetic peak torque of the knee (extension) data, Figure S4. Q–Q plots are used to graphically assess normality of the determination test (DT) data, Table S1. Descriptive statistics, percentage change (% change), and 95% CIs by groups.
