# Supplementary material for: Short-Term Recovery Interventions Using Cryosauna, Cold-Water Immersion, and Foam Rolling in Mixed Martial Arts Athletes: A Polish Pilot Study
Source: Sports (Basel). 2026 Jun 12;14(6):244. doi: 10.3390/sports14060244 (PMC13306673; doi:10.3390/sports14060244)
Supplement: Supplementary file 1 [file sports-14-00244-s001.zip › sports-4239157-Figurs S1-S4.pdf]

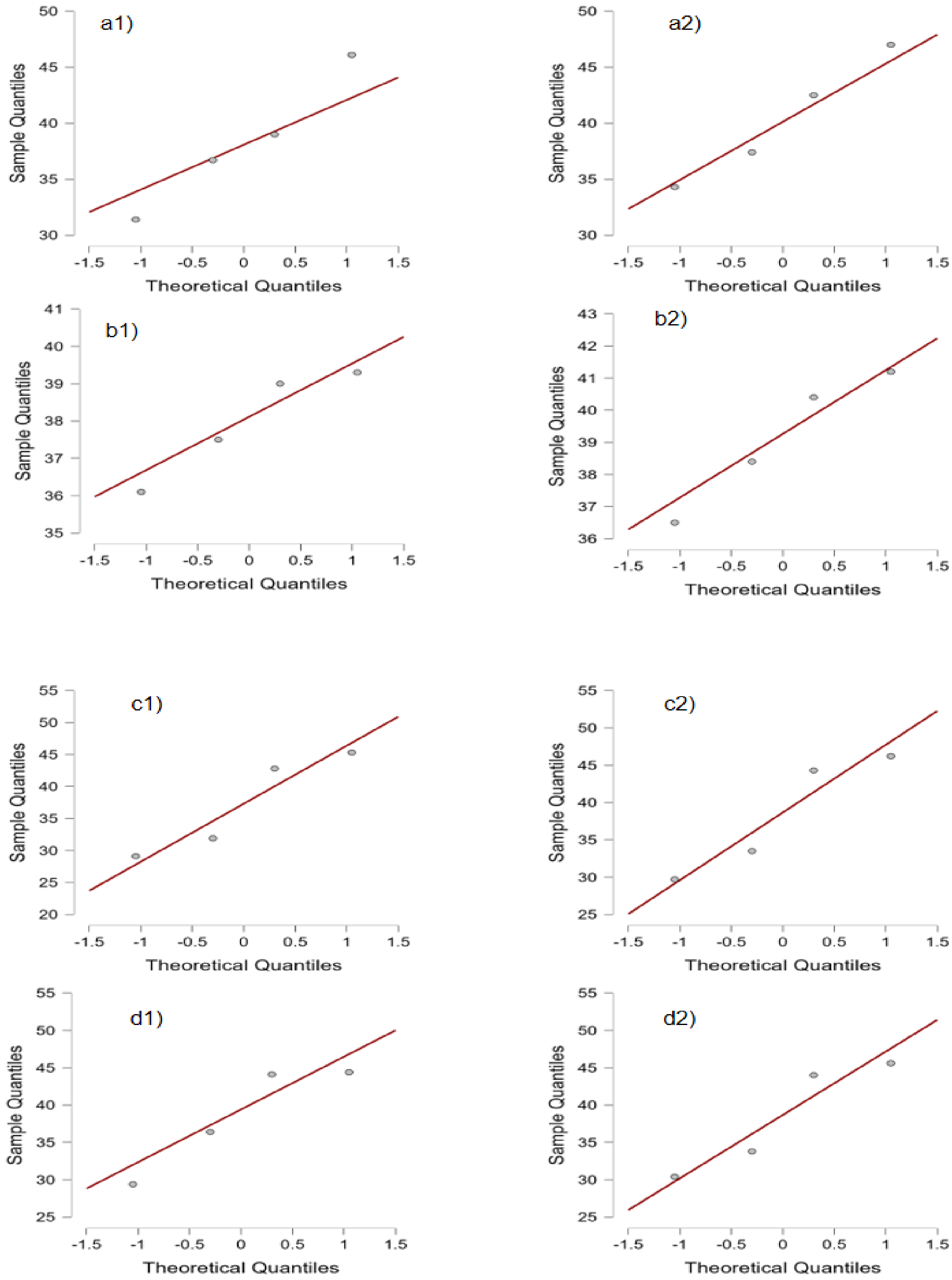

Supplementary Figure S1. Q–Q plots for assessing the normality of countermovement jump (CMJ) data. Panels a1 and a2 show the cryosauna pre- and post-test values, respectively; b1 and b2 show the cold water immersion pre- and post-test values; c1 and c2 show the foam rolling pre- and post-test values; and d1 and d2 show the control pre- and post-test values.

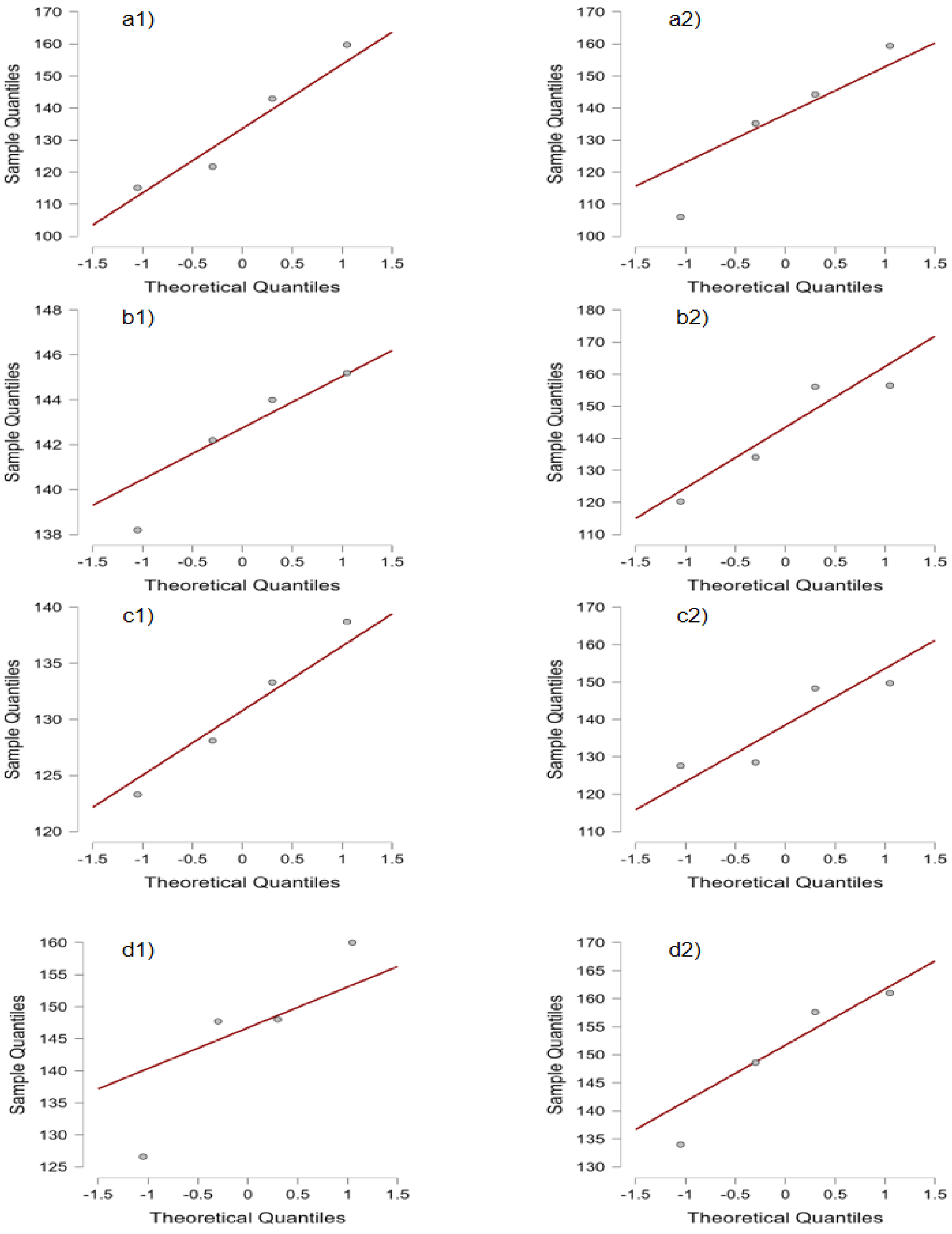

Supplementary Figure S2. Q–Q plots are used for the graphical assessment of normality for the isokinetic peak torque of the knee (flexion) data. Panels a1 and a2 show the cryosauna pre- and post-test values, respectively; b1 and b2 show the cold water immersion pre- and post-test values; c1 and c2 show the foam rolling pre- and post-test values; and d1 and d2 show the control pre- and post-test values.

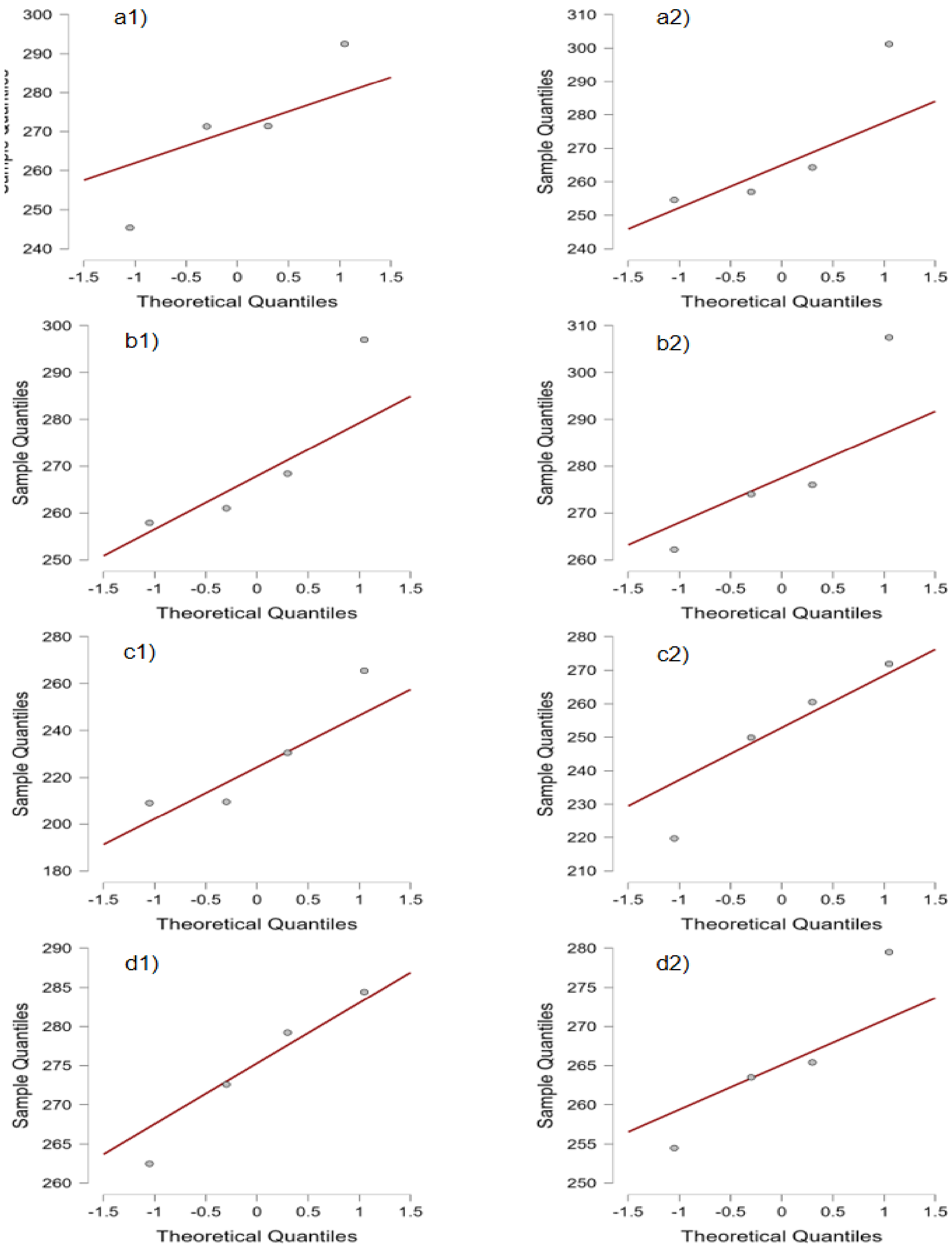

Supplementary Figure S3. Q–Q plots are used for the graphical assessment of normality for the isokinetic peak torque of the knee (extension) data. Panels a1 and a2 show the cryosauna pre- and post-test values, respectively; b1 and b2 show the cold water immersion pre- and post-test values; c1 and c2 show the foam rolling pre- and post-test values; and d1 and d2 show the control pre- and post-test values.

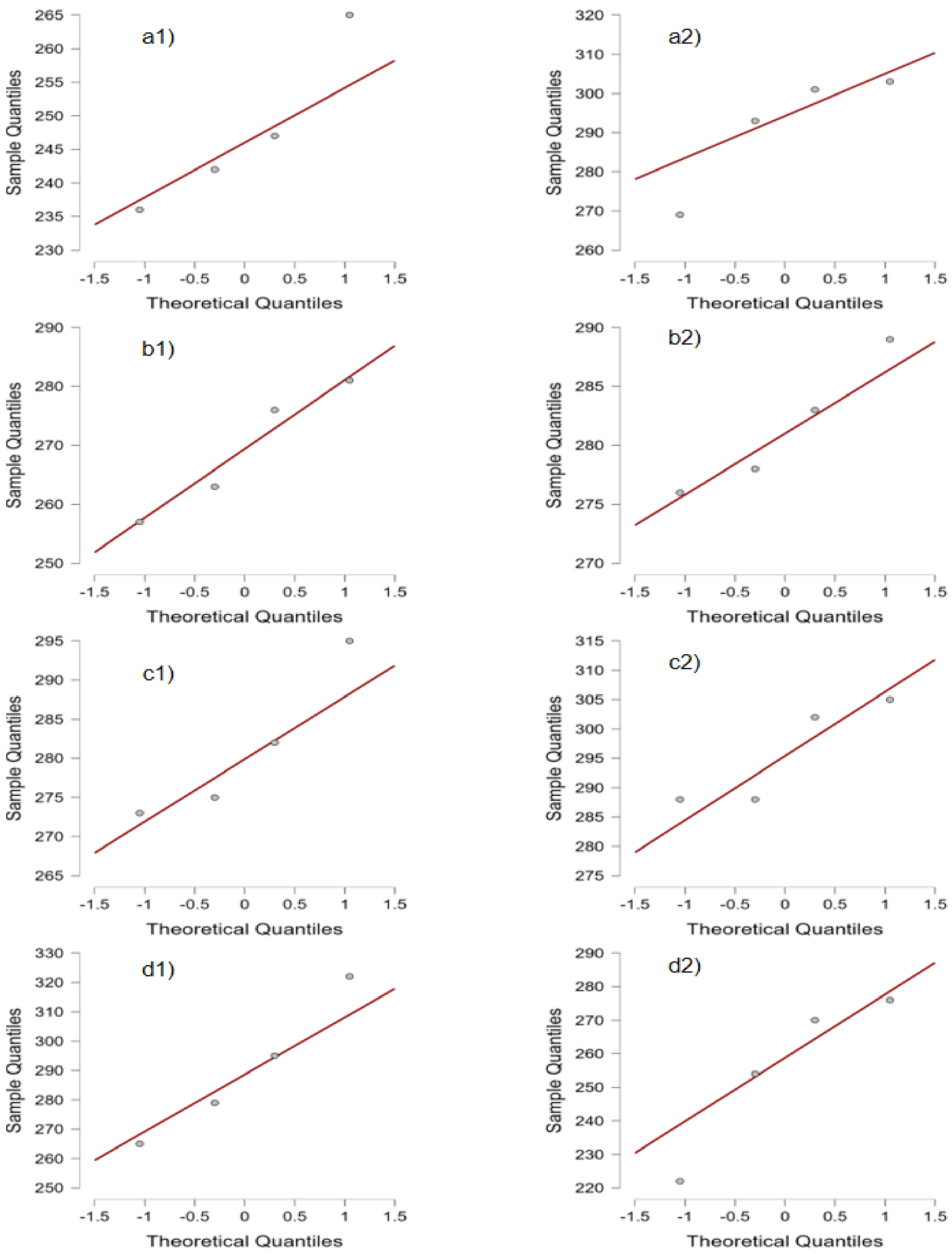

Supplementary Figure S4. Q–Q plots are used to graphically assess normality of the determination test (DT) data. Panels a1 and a2 show the cryosauna pre- and post-test values, respectively; b1 and b2 show the cold water immersion pre- and post-test values; c1 and c2 show the foam rolling pre- and post-test values; and d1 and d2 show the control pre- and post-test values.
