# Supplementary material for: Short-Term Recovery Interventions Using Cryosauna, Cold-Water Immersion, and Foam Rolling in Mixed Martial Arts Athletes: A Polish Pilot Study
Source: Sports (Basel). 2026 Jun 12;14(6):244. doi: 10.3390/sports14060244 (PMC13306673; doi:10.3390/sports14060244)
Supplement: Supplementary file 1 [file sports-14-00244-s001.zip › sports-4239157-Table S1.pdf]

Table S1. Descriptive statistics, percentage change (% change), and 95% CIs by groups.

Parameters	Test phase	Cryo n=4 [95% CI]	% Change	CWI n=4 [95% CI]	% Change	FR n=4 [95% CI]	% Change	Control n=4 [95% CI]	% Change
CMJ (cm)	Pre	38.30 ± 6.09 [28.59, 48.00]	5.22	37.97 ± 1.47 [35.62, 40.32]	3.02	37.27 ± 7.92 [24.59, 49.96]	3.08	38.57 ± 7.15 [27.19, 49.95]	-0.33
	Post	40.30 ± 5.60 [31.38, 49.21]		39.12 ± 2.10 [35.76, 42.48]		38.42 ± 8.06 [25.58, 51.26]		38.45 ± 7.49 [26.53, 50.37]	
Isokinetic knee flex (N.m)	Pre	134.85 ± 20.37 [102.43, 167.27]	0.96	142.40 ± 3.05 [137.53, 147.26]	-0.42	130.85 ± 6.63 [120.28, 141.41]	5.80	145.57 ± 13.88 [123.47, 167.67]	3.22
	Post	136.20 ± 22.47 [100.43, 171.96]		141.75 ± 17.72 [113.55, 169.94]		138.52 ± 12.11 [119.24, 157.80]		150.30 ± 12.06 [131.10, 169.49]	
Isokinetic knee ext (N.m)	Pre	270.15 ± 19.27 [239.47, 300.82]	-0.33	271.07 ± 17.83 [242.69, 299.45]	3.24	228.67 ± 26.49 [186.51, 270.83]	9.52	274.67 ± 9.44 [259.64, 289.70]	-3.27
	Post	269.27 ± 21.67 [234.77, 303.77]		279.92 ± 19.36 [249.11, 310.74]		250.50 ± 22.41 [214.83, 286.16]		265.72 ± 10.34 [249.27, 282.18]	
DT (AU)	Pre	247.50 ± 12.50 [227.60, 267.39]	17.77	269.25 ± 11.14 [251.51, 286.98]	4.53	281.25 ± 9.94 [265.42, 297.07]	5.15	290.25 ± 24.45 [251.33, 329.17]	-11.98
	Post	291.50 ± 15.61 [266.66, 316.39]		281.50 ± 5.80 [272.26, 290.73]		295.75 ± 9.03 [281.37, 310.12]		255.50 ± 24.18 [217.01, 293.98]	

Note: values are means ± standard deviation. CI: confidence interval; CMJ: countermovement jump; cm: centimeter; flex: flexion; ext: extension; N.m: newton meter; DT: determination test; AU: arbitrary units.
